# Occurrence of Hospital-Associated Thrombosis in the Setting of Current Thromboprophylaxis Strategies: An Observational Cross-Sectional Study

**DOI:** 10.1055/a-2137-9531

**Published:** 2023-09-27

**Authors:** Chantal Visser, Marieke J. H. A. Kruip, Janet Brantsma-Van der Graaf, Eric E. van Thiel, Mark-David Levin, Peter E. Westerweel

**Affiliations:** 1 Department of Hematology, Erasmus MC, Erasmus University Medical Center Rotterdam, Rotterdam, The Netherlands; 2 Department of Internal Medicine, Albert Schweitzer Hospital Dordrecht, Dordrecht, The Netherlands; 3 Department of Pulmonology, Albert Schweitzer Hospital Dordrecht, Dordrecht, The Netherlands

## Research Letter

Hospital-associated thrombosis (HAT) is a common potentially preventable cause of morbidity and mortality. 1 2 3 4 HAT can be effectively lowered by thromboprophylaxis. 5 6 7 Studies have shown that thromboprophylaxis guidelines remain underutilized and a significant proportion of hospitalized patients do not receive the recommended thromboprophylaxis. 8 9 Subsequently, most HAT improvement programs have focused on increasing the adherence to thromboprophylaxis guidelines. 10 11 Low adherence to guidelines is commonly believed to cause the development of HAT. Nevertheless, it is important to realize that the effectiveness of thromboprophylaxis is not 100%, 5 6 7 so adequately following thromboprophylaxis guidelines will not always prevent HAT. 12 Therefore, we aimed to describe the proportion of HAT patients in whom the thromboprophylaxis strategies were correctly applied.

In this cross-sectional observational study, we used data from a large Dutch teaching hospital. We included all adult patients who visited the outpatient department with a diagnosis of pulmonary embolism (PE) and/or deep vein thrombosis (DVT) as a venous thrombotic event (VTE), between January 1, 2016 and December 31, 2018. The regional care pathway for VTE involved minimally one scheduled visit to the outpatient department, during which the patients' treatment options and duration and risk profiles were systematically discussed and recorded. The collected information includes concomitant risk factors for VTE, recent surgical procedures and hospital admission and, if applicable, the administration of thromboprophylaxis during hospitalization.

Our main study outcome was the proportion of HAT patients in whom the thromboprophylaxis protocol was correctly applied. We defined HAT as the occurrence of radiologically confirmed DVT and/or PE within 3 months following surgical interventions, including casting and/or surgery, hospital admission (>48 hours), or a combination of both. We defined thromboprophylaxis as any pharmacological thromboprophylaxis received after surgical intervention or during hospital admission. The local thromboprophylaxis protocol for hospitalized patients is similar to the national guideline. 13 The dosage of pharmacological thromboprophylaxis is determined based on patient-specific factors, including weight, renal function, and the risk of thrombosis. Risk assessment includes various criteria such as the type of procedure (low, moderate, and high risk), hospital admission reason, same-day treatment, and additional risk factors included in the Padua prediction score 14 and Caprini score, 15 as outlined in Supplementary Table S1 and Supplementary File S1 ( Supplementary Material ). Based on this risk stratification assessment, patients with HAT were categorized as either low risk (not requiring thromboprophylaxis) or high risk (requiring thromboprophylaxis).

We analyzed the occurrence of HAT by stratifying events based on timing and type of hospital visit: medical patients (hospital admission >48 hours), short-term admitted (surgical procedure) and hospitalized surgical patients (combination of hospital admission >48 hours and surgical procedure). Descriptive statistics were expressed as median with interquartile range (IQR), mean with standard deviation, or counts with percentages (%). Data were compared using the independent *t* -test, Mann–Whitney U-test, chi-square test, or Fisher's exact test depending on the type and the distribution of the data. The 95% confidence intervals (95% CIs) for percentages were calculated using the Clopper–Pearson method. Statistical analyses were performed with IBM SPSS statistics version 25.

Of the 1,164 patients who visited the outpatient department, 187 patients (16.1%, 95% CI: 14.0–18.3%) experienced a HAT. These HAT patients were slightly older (67 [51–77] vs. 64 [51–74], *p* = 0.229) and were more often female (100/187 (53.5%) vs. 466/977 (47.7%), *p* = 0.147) compared to patients without HAT, although these differences were not statistically significantly ( Table 1 ). Of the 187 HAT patients, 75 (40.1%) had undergone surgical procedures, 38/187 (20.3%) had been admitted to the hospital, and 74/187 (39.6%) had a combination of both. Other baseline characteristics are summarized in Table 1 .

**Table 1:** Baseline characteristics

	Patients with HAT ( *n* = 187)				Patients without HAT ( *n* = 971)
	All	Short-term admitted surgical patients ( *n* = 75) a	Medical patients ( *n* = 38) b	Hospital-admitted surgical patients ( *n* = 74) c	
Baseline characteristics					
Age (median, IQR)	67.0 (51.0–77.0)	57.0 (46.0–68.0)	73.0 (60.5–80.0)	69.0 (55.8–77.0)	64.0 (51.0–74.0)
Male ( *n* , %)	87 (46.5)	40 (53.3)	13 (34.2)	34 (45.9)	511 (52.3)
Type of VTE					
DVT ( *n* , %)	80 (42.8)	42 (56.0)	17 (44.7)	21 (28.4)	444 (45.4)
PE ( *n* , %)	100 (53.5)	29 (38.7)	20 (52.6)	51 (68.9)	479 (49.0)
Both ( *n* ,%)	7 (3.7)	4 (5.3)	1 (2.6)	2 (2.7)	54 (5.5)
Risk factors					
VTE history ( *n* ,%)	29 (15.5)	11 (14.7)	6 (15.8)	12 (16.2)	250 (25.6)
Immobility ( *n* ,%)	74 (39.6)	36 (48.0)	21 (55.3)	17 (23.0)	83 (8.5)
Malignancy ( *n* ,%)	52 (27.8)	15 (20.0)	10 (26.3)	27 (36.5)	162 (16.6)
Family history ( *n* ,%)	25 (13.4)	17 (22.7)	1 (2.6)	7 (9.5)	159 (16.3)
Oral contraceptives ( *n* ,%)	13 (7.0)	6 (8.0)	2 (5.3)	5 (6.8)	115 (11.8)
Pregnancy ( *n* ,%)	3 (1.6)	1 (1.3)	1 (2.6)	1 (1.4)	12 (1.2)
Extended travel ( *n* ,%)	1 (0.5)	1 (1.3)	0 (0.0)	0 (0.0)	83 (8.5)
Scores					
Padua score (median, IQR) d	5.0 (4.0–6.0)	–	4.0 (3.0–6.0)	6.0 (5.0–7.0)	
Caprini score (median, IQR) e	7.0 (5.0–10.0)	–	–	7.0 (5.0–10.0)	
Hospital admission and thromboprophylaxis					
Preadmission therapeutic anticoagulation ( *n* , %)	10 (5.3)	1 (1.3)	4 (10.5)	5 (6.8)	
Thromboprophylaxis ( *n* , %)	123 (65.8)	29 (38.7)	28 (73.7)	66 (89.2)	
2,500 IE ( *n* ,%)	32 (17.1)	6 (8.0)	13 (34.2)	13 (17.6)	
5,000 IE ( *n* , %)	75 (40.1)	19 (25.3)	11 (28.9)	45 (60.8)	
7,500 IE ( *n* ,%)	1 (0.5)	0 (0.0)	0 (0.0)	1 (1.4)	
Duration of thromboprophylaxis in days (median, IQR)	8.0 (5.0–27.0)	21.0 (7.0–42.0)	8.5 (6.3–19.0)	7.0 (5.0–18.0)	
Length of stay (median, IQR)	4.0 (0.0–9.0)	–	8.0 (5.0–13.3)	7.5 (4.0–13.3)	

Abbreviations: DVT: deep vein thrombosis; HAT: hospital associated thrombosis; IQR: Interquartile range; PE: pulmonary embolism; VTE: venous thromboembolic event.

a Short-term-admitted surgical patients is defined as patients who received a surgical procedure and were admitted <48 hours.

b Medical patients is defined as patients who were admitted to the hospital more than 48 hours.

c Hospital-admitted surgical patients is defined as patients who were admitted to the hospital more than 48 hours and received a surgical procedure.

d The Padua score was only calculated for the surgical and nonsurgical hospitalized patients.

e The Caprini score was only calculated for the hospital-admitted surgical patients.

The appropriate thromboprophylaxis strategy was correctly applied in 153 of the 187 HAT patients (81.8%, 95% CI: 75.5–87.1%) ( Table 2 ). All patients received low-molecular-weight heparin based on their weight and renal function. Among the 120 high-risk patients, 104 (87.5%) received thromboprophylaxis. Of the 67 low-risk patients, 48 (71.6%) did not receive thromboprophylaxis. The proportion of HAT patients in whom the thromboprophylaxis strategy was correctly applied did not differ between medical and surgical patients ( Table 2 ). Although low-risk patients did not differ in terms of gender or the percentages of concomitant risk factors compared to high-risk patients, they were younger (59.0 (47.0–70.0) vs. 69.0 (55.0–78.0), *p* = 0.024). Notably, the proportion of high-risk patients who developed HAT despite receiving thromboprophylaxis was the highest in the hospitalized surgical patients. In contrast, the short-term admitted surgical patients were more often low-risk patients compared to the other two groups.

**Table 2:** Thromboprophylaxis given in patients with high and low risk of hospital-associated thrombosis

	High-risk patients ( *n* = 120)	Low-risk patients ( *n* = 67)	*p* -Value
	All patients with hospital-associated thrombosis a ( *n* = 187)		<0.001
Thromboprophylaxis (+) ( *n* ,%) b	104 (87.5)	17 (26.9)	<0.001
Thromboprophylaxis (−) ( *n* , %)	12 (10.0)	48 (71.6)	<0.001
Thromboprophylaxis (unknown) ( *n* , %)	3 (2.5)	1 (1.5)	1.000
	Short-term admitted surgical patients c ( *n* = 75)		<0.001
Thromboprophylaxis (+) ( *n* , %)	24 (72.7)	5 (11.9)	<0.001
Thromboprophylaxis (−) ( *n* , %)	7 (21.2)	37 (88.1)	<0.001
Thromboprophylaxis (unknown) ( *n* , %)	2 (6.1)	0 (2.3)	1.000
	Medical patients d ( *n* = 38)		0.008
Thromboprophylaxis (+) ( *n* , %)	25 (89.3)	3 (30.0)	0.003
Thromboprophylaxis (−) ( *n* , %)	3 (10.7)	6 (60.0)	0.020
Thromboprophylaxis (unknown) ( *n* , %)	0 (0.0)	2 (20.0)	0.181
	Hospitalized surgical patients e ( *n* = 74)		0.005
Thromboprophylaxis (+) ( *n* , %)	55 (94.9)	10 (66.7)	0.020
Thromboprophylaxis (−) ( *n* , %)	2 (3.4)	5 (33.3)	0.005
Thromboprophylaxis (unknown) ( *n* , %)	2 (3.4)	0 (0.0)	1.000

Note: *p* -Value was calculated using chi-square test and corrected with a Bonferroni correction.

a According to the local protocol.

b Thromboprophylaxis is defined as any pharmacological thromboprophylaxis after hospital admission or surgical procedure.

c Short-term admitted surgical patients is defined as patients who received a surgical procedure and were admitted <48 hours.

d Medical patients is defined as patients who were admitted to the hospital more than 48 hours.

e Hospitalized surgical patients is defined as patients who were admitted to the hospital more than 48 hours and received a surgical procedure.

**Fig. 1:**
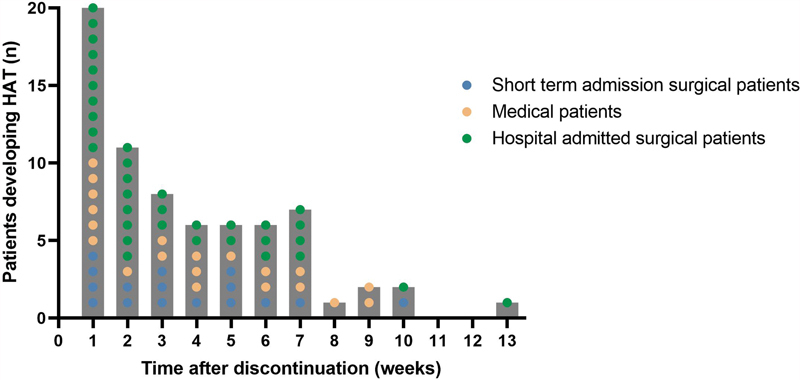
**Hospital-associated thrombosis after discontinuation of thromboprophylaxis.** Dotplot of the number of medical, short-term, and hospital-admitted surgical patients who developed a hospital-associated thrombosis after discontinuation of thromboprophylaxis stratified per week.

Most patients developed HAT after discontinuing thromboprophylaxis (63.9%, 95% CI: 54.7–72.4%), with a median [IQR] of 2.1 (0.7–5.1) weeks after discontinuation ( Fig. 1 ). In half of the high-risk medical and surgical hospitalized patients, thromboprophylaxis was not continued after discharge (40/80, 50.0%). Of all the hospitalized patients, 78/112 (69.6%) developed a HAT after discharge, with a median of 19.0 (5.3–43.8) days. Interestingly, the majority of medical patients developed HAT after discontinuation (20/28, 71.4%), particularly following the discontinuation of thromboprophylaxis before discharge (12/20 (60.0%)).

In this study, we observed a high proportion of HAT patients in whom the thromboprophylaxis strategies were mostly correctly applied. More than half of the HAT patients developed a VTE despite receiving a period of thromboprophylaxis. Other observational studies have found similar proportions of patients developing venous thrombosis despite receiving thromboprophylaxis. 16 17 18 The development of HAT despite thromboprophylaxis has also occurred in investigational studies. 5 6 7 19 These observations, in combination with our results, give some contrast to the common perception of physicians and patients that HAT only develops due to omitted thromboprophylaxis.

A major strength of our study is the use of real-world data from a large cohort of consecutive outpatient patients and is, therefore, representative of general clinical practice. Our study also has limitations. It specifically focusses on patients who have experienced an event and thereby lacks information about patients who did not receive thromboprophylaxis or discontinued thromboprophylaxis without experiencing an event. In addition, we could not include asymptomatic or fatal VTE nor patients who were not referred to the outpatient department. However, most patients with symptoms or diagnosis of VTE living in the area are referred to this hospital for diagnosis and treatment of VTE, especially when patients have received treatment in the hospital. We assume that these misclassifications of VTE are independent of whether thromboprophylaxis was correctly applied.

Apart from reinforcing adherence to guidelines, other optimization strategies for preventing HAT can be explored. Possible solutions might include improved identification of high-risk patients, extended duration of thromboprophylaxis, and the development of more effective thromboprophylactic drugs. In our study, 70 of the 187 HAT patients developed a HAT despite being classified as low risk. This suggests that the current risk assessment, which incorporates factors such as the type of procedure, reason of hospital admission, and other risk factors, may lack sensitivity in identifying these individuals. Another strategy could involve extending thromboprophylaxis beyond hospital admission. A previous study has shown that 71% of HAT diagnoses are diagnosed after discharge. 20 Similarly, most of our patients developed HAT after thromboprophylaxis was discontinued. A recent randomized controlled trial in selected high-risk patients reported that extended thromboprophylaxis was not associated with their primary composite outcome of symptomatic nonfatal VTE and fatal VTE. However, extended thromboprophylaxis resulted in a lower incidence of nonfatal symptomatic VTE as a secondary outcome. 21 Therefore, extended thromboprophylaxis may be beneficial for selected high-risk patients in preventing HAT. Finally, the development of thromboprophylactic drugs with improved efficacy and safety profiles might be a solution. Factor XI inhibitors, for instance, have shown potential in effectively preventing VTE without increasing the risk of bleeding in phase 2 trials. Several phase 3 trials are currently studying whether these inhibitors are indeed more effective in preventing VTE without a comparable risk of bleeding. 22 23 If proven successful, these drugs could help mitigate the high prevalence of HAT among hospitalized patients.
