# Supplementary material for: Occurrence of Hospital-Associated Thrombosis in the Setting of Current Thromboprophylaxis Strategies: An Observational Cross-Sectional Study
Source: TH Open. 2023 Sep 27;7(3):e280–4. doi: 10.1055/a-2137-9531 (PMC10533216; doi:10.1055/a-2137-9531)
Supplement: Supplementary file 1 — Supplementary Material [file 10-1055-a-2137-9531-s23040013.pdf]

Supplemental Material

Supplementary Table S1 Thromboprophylaxis based on VTE risk

	Procedure	Reason for hospital admission	Other risk factors	Padua score
Low risk<br>No pharmacological thromboprophylaxis	Laparoscopic surgery <45 minutes<br>Appendectomy<br>Transurethral resection of the prostate (TURP)<br>Inguinal hernia repair<br>Vascular access surgery (such as dialysis shunt catheters)<br>Carotid endarterectomy<br>Nonmalignant breast, head and neck surgery			Padua score 0[a]<br>Padua score <4[a]
Moderate risk<br>Dalteparin 2,500 IE subcutaneous once daily	Bowel resection<br>Gastric surgery<br>Open cholecystectomy<br>Amputation of the leg (above and below the knee amputations)<br>Surgery for breast cancer<br>Major neurosurgical procedures<br>Day surgery with general anaesthesia	Acute phase of ischemic CVA with immobility due to the paresis of the leg<br>ICU patients<br>Heart failure<br>Severe respiratory diseases<br>Severe acute infections or active cancer	Bed rest >3 days<br>Heart failure<br>Severe respiratory diseases<br>Severe acute infections or active cancer	Padua score <4, but other risk factors not mentioned in the Padua score (or in the section of other risk factors)<br>Padua score $\geq$ 4
High risk<br>Dalteparin 5,000 IE subcutaneous once daily	Oncologic resection of the abdomen or thorax<br>Major vascular surgery<br>Pneumonectomy<br>Pelvic surgery<br>Gynaecological malignancy (consider treatment regimen 2b; high risk extended thromboprophylaxis)<br>Abdominal wall reconstruction<br>Bariatric surgery		Weight > 100 kg in patients with moderate risk	Padua score $\geq$ 4 and other risk factors
High risk extended thromboprophylaxis<br>Dalteparin 5,000 IE subcutaneous once a day 28 to 35 days	Knee arthroplasty<br>Hip arthroplasty<br>Traumatic hip fracture	Traumatic hip fracture		Padua score $\geq$ 4 and other risk factors which indicate extended thromboprophylaxis

Abbreviations: CVA, cerebrovascular accident; ICU, intensive care unit; VTE, venous thromboembolism.
[a]If the patient cannot be classified based on type of procedure, hospital admission, or other risk factors.

Supplementary File S1 Thromboprophylaxis protocol of Albert Schweitzer Hospital

This thromboprophylaxis protocol was translated from Dutch to English.

Pathophysiology

There is an increased risk of venous thrombosis in clinical patients. Thromboprophylaxis can be administered to decrease this risk. This hospital protocol states when thromboprophylaxis is needed and which type of thromboprophylaxis should be administered (duration, type, and dose).

Scope

This protocol is a hospital-wide medical treatment protocol. This protocol applies to every medical specialist and house officer who administers thromboprophylaxis to hospitalized patients and patients visiting the outpatient department of the Albert Schweitzer Hospital. This protocol applies to surgical and medical patients. A pediatrician should be consulted if the patient is younger than 16 years.

 © 2023. The Author(s).

Treatment Regimen

Thromboprophylaxis should be administered following one of the below-mentioned treatment regimens based on the risk of thrombosis of the patient.

Dalteparin should be given postoperative on the evening of the operation at 22 hours. Patients of the day treatment who have been given general or regional anesthesia of the leg are the exceptions. In these patients, dalteparin should be given before the moment of discharge. In the case of spinal or epidural anesthesia, four hours should be between the block and the administration of thromboprophylaxis.

The lead physician is responsible for the administration of thromboprophylaxis. One can consult the anesthesiologist if needed. In most cases, patients of the day treatment do not have an indication for thromboprophylaxis. *If these patients have an indication of thromboprophylaxis, do not forget to give them the prescription before discharge.*

Treatment regimen 0

No thromboprophylaxis

Treatment regimen 1a

Dalteparin 2,500 IE subcutaneous once daily. If a patient weighs more than 100 kg → 5,000 IE subcutaneous once a day.

Treatment regimen 2a

Dalteparin 5,000 IE subcutaneous once daily.

Treatment regimen 2b

Dalteparin 5,000 IE subcutaneous once a day 28 to 35 days.

Footnote a

Thromboprophylaxis should be stopped at the moment of discharge, except when there is a need for extended thromboprophylaxis. In this case, a prescription should be given to the patient. Dalteparin is the first choice of thromboprophylaxis, but a direct oral anticoagulant (DOAC) or fondaparinux can also be used in case of an elective hip or knee arthroplasty.

The following protocol should be followed:

- Apixaban 2.5 mg twice daily, starting 24 hours after surgery.
- Dabigatran 150 mg once daily, starting 24 hours after surgery.
- Fondaparinux 2.5 mg subcutaneous once daily, starting minimally 6 hours after surgery.

In the case of spinal or epidural anesthesia, a 24-hour gap should be between the block and the administration of a DOAC and 4 hours between the block and the administration of low-molecular-weight heparin (LMWH).

In surgical patients, the hospital protocol of perioperative management of anticoagulation should also be followed.

Specific Patient Populations

Risk of Thrombosis

In this paragraph, several categories of venous thromboembolism (VTE) risks are described. Thromboprophylaxis is administered according to the above-mentioned treatment regimens depending on the risk of VTE. If a patient cannot be categorized based on one of these categories, the VTE risk should be calculated using the Padua prediction score (paragraph 6) as mentioned by the Dutch national guideline.

Treatment Regimen 0

- Laparoscopic surgery <45 minutes
- Appendectomy
- Transurethral resection of the prostate (TURP)
- Inguinal hernia repair
- Vascular access surgery (such as dialysis shunt catheters)
- Carotid endarterectomy
- Nonmalignant breast, head, and neck surgery.

Treatment Regimen 1a

- Bowel resection
- Gastric surgery
- Open cholecystectomy
- Amputation of the leg (above and below the knee amputations)
- Surgery for breast cancer
- Major neurosurgical procedures

 © 2023. The Author(s).

- Day surgery with general anesthesia
- Heart failure
- Severe respiratory diseases
- Severe acute infections or active cancer
- Bed rest >3 days
- Intensive care unit patients
- Acute phase of ischemic CVA (cerebrovascular accident) with immobility due to the paresis of the leg.

Treatment Regimen 2a

- Oncologic resection abdomen/thorax
- Major vascular surgery
- Pneumonectomy
- Pelvic surgery
- Gynecological malignancy (consider treatment regimen 2b)
- Abdominal wall reconstruction
- Bariatric surgery.

Treatment Regimen 2b

- Knee arthroplasty
- Hip arthroplasty
- Traumatic hip fracture
- In the case of gynecological malignancy, extended thromboprophylaxis of 6 weeks should be considered.

Every other patient: calculate the Padua prediction risk score. If the Padua prediction score is >4, thromboprophylaxis should be considered.

Pregnancy and Postpartum

Preconception Care

Patients with a history of VTE or thrombophilia should be referred to the outpatient department of obstetrics to receive preconception recommendations.

During Pregnancy and Postpartum

Thromboprophylaxis during pregnancy and postpartum should be conformed to the new guideline of the NVOG (the Dutch Society for Obstetrics and Gynaecology). This guideline can be found using the url: www.nvog.nl>vakinformatie>richtlijnen>professionals>perinatologie: "**Diagnostiek, behandeling en preventie van trombo-embolie in de zwangerschap.**"

Partus

The LMWH should be interrupted when the partus is starting to reduce the risk of postpartum hemorrhage. LMWH can be restarted the day after the partus if the risk of bleeding is low.

Caesarean Section

Prophylactic LMWH: stop 10 hours before the spinal anesthesia and restart 4 hours after the block in consultation with the surgeon.

Therapeutic LMWH: stop 24 hours before the section. The therapeutic dose can be restarted 24 hours after the section in consultation with the surgeon. One should administer prophylaxis LMWH. It is best to give the therapeutic LMWH in the morning. This way, the last dose can be given the morning of the day before the section.

Spinal or Epidural Anesthesia in Pregnant Women

Prophylactic LMWH: an epidural anesthesia can be given 10 to 12 hours after the last dose of LMWH.

Therapeutic LMWH is a contraindication for epidural or spinal anesthesia, and a minimum period of 24 hours is needed between the last dose of LMWH and the block. The minimal time between the block and the next LMWH dose is 24 hours. Prophylactic LMWH should be given (as mentioned above).

Children Aged 15 Years or Younger

Venous thrombosis in children is very rare. One should question whether prophylaxis is needed in light of the low incidences of VTE and the specific social context in children.

Children aged 12 to 16 are lawfully allowed to refuse thromboprophylaxis, but the responsible guardian should be informed about the possible consequences. From 16 years onwards, children are lawfully fully autonomous.

Guideline:

- Children <16 years: consult the pediatrician.
- 16 years or older: use this hospital protocol and the Padua prediction score.
- One should always write down the decision of thromboprophylaxis in children with arguments in the electronic patient file.

Padua Prediction Score

- Active cancer: +3
- Previous VTE: +3
- Reduced mobility (>3 days): +3
- Already known thrombophilia: +3
- Recent (<1 month) trauma and/or surgery: +2
- Elderly age (>70 years): +1
- Heart failure/COPD: +1
- Acute myocardial infarction and/or ischemic stroke: +1
- Acute infection and/or rheumatologic disorder: +1
- Body mass index >30: +1
- Ongoing hormonal treatment (oral anticonception or suppletion): +1

0 points: pharmacologic prophylaxis is not indicated.
<4 points: the need for pharmacologic thromboprophylaxis is assessed per patient.
Be cautious with the use of pharmacologic prophylaxis.
>4 points: pharmacologic thromboprophylaxis should be administered.

Complications

The use of dalteparin can result in local subcutaneous hematoma or skin irritation. The risk of bleeding is increased at higher doses of dalteparin or DOACs.

Exceptions

Patients with a history of Heparin-Induced Thrombocytopenia and Thrombosis (HITT) after dalteparin should be administered alternatives such as danaparoid or DOAC. The time intervals and possibilities of spinal and epidural anesthesia are also different in these patients. Please consult the hospital protocol of perioperative management.

Discharge and Outpatient Visits

It is essential to extend the prophylaxis in case of knee and hip arthroplasty and traumatic hip fractures at the moment of discharge. In the case of pelvic surgery due to gynecological cancers, the thromboprophylaxis should be extended to 6 weeks. Extended thromboprophylaxis should be considered if the patient is immobile at discharge.

 © 2023. The Author(s).
